# Guidance for Fecal Microbiota Transplantation Trials in Ulcerative Colitis: The Second ROME Consensus Conference

**DOI:** 10.1093/ibd/izaf013

**Published:** 2025-02-11

**Authors:** Loris R Lopetuso, Sara Deleu, Pierluigi Puca, Maria Teresa Abreu, Alessandro Armuzzi, Giovanni Barbara, Flavio Caprioli, Siew Chieng, Samuel Paul Costello, Andrea Damiani, Silvio Danese, Federica Del Chierico, Geert D’Haens, Iris Dotan, Federica Facciotti, Gwen Falony, Massimo Claudio Fantini, Gionata Fiorino, Paolo Gionchetti, Lihi Godny, Ailsa Hart, Juozas Kupčinskas, Tariq Iqbal, Lucrezia Laterza, Letizia Lombardini, Nitsan Maharshak, Giovanni Marasco, Luca Masucci, Alfredo Papa, Sudarshan Paramsothy, Valentina Petito, Daniele Piovani, Daniela Pugliese, Lorenza Putignani, Jeroen Raes, Davide Giuseppe Ribaldone, Maurizio Sanguinetti, Edoardo Vincenzo Savarino, Harry Sokol, Stefania Vetrano, Gianluca Ianiro, Giovanni Cammarota, Fabio Cominelli, Theresa T Pizarro, Herbert Tilg, Antonio Gasbarrini, Severine Vermeire, Franco Scaldaferri

**Affiliations:** IBD Unit, CEMAD, Fondazione Policlinico Universitario Agostino Gemelli IRCCS, Rome, Italy; Department of Life Science, Health, and Health Professions, Link Campus University, Rome, Italy; IBD Unit, CEMAD, Fondazione Policlinico Universitario Agostino Gemelli IRCCS, Rome, Italy; Department of Chronic Diseases, Metabolism (CHROMETA), KU Leuven, Leuven, Belgium; Dipartimento di Medicina e Chirurgia Traslazionale, Università Cattolica del Sacro Cuore, Rome, Italy; IBD Unit, CEMAD, Fondazione Policlinico Universitario Agostino Gemelli IRCCS, Rome, Italy; Dipartimento di Medicina e Chirurgia Traslazionale, Università Cattolica del Sacro Cuore, Rome, Italy; Division of Gastroenterology, Department of Medicine, Crohn’s and Colitis Center, University of Miami - Miller School of Medicine, Miami, FL, USA; IBD Unit, IRCCS Humanitas Research Hospital, Milan, Italy; Department of Biomedical Sciences, Humanitas University, Milan, Italy; Department of Medical and Surgical Sciences, University of Bologna, Bologna, Rome, Italy; IRCCS Azienda Ospedaliero Universitaria Di Bologna, Bologna, Italy; Gastroenterology and Endoscopy Unit, Fondazione IRCCS Ca’ Granda Ospedale Maggiore Policlinico, Milan, Italy; Department of Pathophysiology and Transplantation, Università degli Studi di Milano, Milan, Italy; Department of Medicine and Therapeutics, Faculty of Medicine, The Chinese University of Hong Kong, Hong Kong, China; Department of Gastroenterology, The Queen Elizabeth Hospital, Adelaide, SA, Australia; Real World Data Facility, Gemelli Generator, Fondazione Policlinico Universitario Agostino Gemelli IRCCS, Rome, Italy; Department of Gastroenterology and Digestive Endoscopy, IRCCS San Raffaele Hospital and Vita-Salute San Raffaele University, Milan, Italy; Immunology, Rheumatology and Infectious Diseases Research Area, Unit of Human Microbiome, Bambino Gesù Children’s Hospital IRCCS, Rome, Italy; Department of Gastroenterology and Hepatology, Amsterdam University Medical Center, Amsterdam, The Netherlands; Division of Gastroenterology, Rabin Medical Center, Petah-Tikva, Israel; Dipartimento di Biotecnologie e Bioscienze, Università di Milano-Bicocca, Milan, Italy; Institute of Medical Microbiology and Hygiene and Research Center for Immunotherapy (FZI), University Medical Center of the Johannes Gutenberg-University Mainz, Mainz, Germany; Department of Medical Sciences and Public Health, University of Cagliari, Cagliari, Italy; Gastroenterology Unit, Azienda Ospedaliero-Universitaria di Cagliari, Cagliari, Italy; IBD Unit, San Camillo-Forlanini Hospital, Rome, Italy; Department of Medical and Surgical Sciences, University of Bologna, Bologna, Italy; IBD Unit, IRCCS Azienda Ospedaliero Universitaria di Bologna, Bologna,, Italy; Division of Gastroenterology, Rabin Medical Center, Petah-Tikva, Israel; IBD Unit, St Mark’s Hospital, Harrow, Middlesex, UK; Department of Gastroenterology, Lithuanian University of Health Sciences, Kaunas, Lithuania; Microbiome Treatment Center, University of Birmingham, Birmingham, UK; IBD Unit, CEMAD, Fondazione Policlinico Universitario Agostino Gemelli IRCCS, Rome, Italy; Dipartimento di Medicina e Chirurgia Traslazionale, Università Cattolica del Sacro Cuore, Rome, Italy; Centro Nazionale Trapianti (CNT), Istituto Superiore di Sanità, Rome, Italy; Division of Gastroenterology, Rabin Medical Center, Petah-Tikva, Israel; IRCCS Azienda Ospedaliero Universitaria Di Bologna, Bologna, Italy; Department of Medical and Surgical Sciences, University of Bologna, Bologna,, Italy; Microbiology Unit, Fondazione Policlinico Universitario Agostino Gemelli IRCCS, Rome, Italy; IBD Unit, CEMAD, Fondazione Policlinico Universitario Agostino Gemelli IRCCS, Rome, Italy; Gastroenterology and Liver Services, Concord Repatriation General Hospital, Sydney, Australia; IBD Unit, CEMAD, Fondazione Policlinico Universitario Agostino Gemelli IRCCS, Rome, Italy; Department of Biomedical Sciences, Humanitas University, Milan, Italy; IBD Unit, CEMAD, Fondazione Policlinico Universitario Agostino Gemelli IRCCS, Rome, Italy; Dipartimento di Medicina e Chirurgia Traslazionale, Università Cattolica del Sacro Cuore, Rome, Italy; Unit of Microbiology and Diagnostic Immunology, Unit of Microbiomics and Research Area of Immunology, Rheumatology and Infectious Diseases, Unit of Human Microbiome, Bambino Gesù Children’s Hospital, IRCCS, Rome, Italy; Department of Microbiology and Immunology, KU Leuven, Leuven, Belgium; Center for Microbiology, VIB, Gent, Belgium; Department of Medical Sciences, Unit of Gastroenterology, University of Turin, Turin, Italy; Microbiology Unit, Fondazione Policlinico Universitario Agostino Gemelli IRCCS, Rome, Italy; Gastroenterology Unit, Azienda Ospedaliera di Padova, Padova, Italy; INSERM, Centre de Recherche Saint-Antoine, CRSA, Sorbonne Université, Paris, France; Department of Gastroenterology, Saint Antoine Hospital, Paris, France; Laboratory of Gastrointestinal Immunopathology, Humanitas Research Hospital, Milan, Italy; Department of Biomedical Sciences, Humanitas University, Milan, Italy; Dipartimento di Medicina e Chirurgia Traslazionale, Università Cattolica del Sacro Cuore, Rome, Italy; Dipartimento di Scienze Mediche e Chirurgiche, UOC di Medicina Interna e Gastroenterologia, Fondazione Policlinico Universitario Agostino Gemelli IRCCS, Rome, Italy; Dipartimento di Scienze Mediche e Chirurgiche, UOC di Medicina Interna e Gastroenterologia, Fondazione Policlinico Universitario Agostino Gemelli IRCCS, Rome, Italy; Division of Gastroenterology and Liver Diseases, Case Western Reserve University School of Medicine, Cleveland, OH, USA; Department of Pathology, Case Western Reserve University School of Medicine, Cleveland, OH, USA; Department of Gastroenterology, Saint Antoine Hospital, Paris, France; Department of Internal Medicine I, Gastroenterology, Hepatology, Endocrinology and Metabolism, Medical University of Innsbruck, Innsbruck, Austria; Dipartimento di Medicina e Chirurgia Traslazionale, Università Cattolica del Sacro Cuore, Rome, Italy; Dipartimento di Scienze Mediche e Chirurgiche, UOC di Medicina Interna e Gastroenterologia, Fondazione Policlinico Universitario Agostino Gemelli IRCCS, Rome, Italy; Department of Chronic Diseases, Metabolism (CHROMETA), KU Leuven, Leuven, Belgium; Department of Gastroenterology and Hepatology, University Hospitals Leuven, Leuven, Belgium; IBD Unit, CEMAD, Fondazione Policlinico Universitario Agostino Gemelli IRCCS, Rome, Italy; Dipartimento di Medicina e Chirurgia Traslazionale, Università Cattolica del Sacro Cuore, Rome, Italy

**Keywords:** fecal microbiota transplantation (FMT), ulcerative colitis (UC), clinical trials, FMT guidelines

## Abstract

**Background:**

Fecal microbiota transplantation (FMT) is emerging as a potential treatment modality for individuals living with inflammatory bowel disease (IBD). Despite its promise, the effectiveness of FMT for treating IBD, particularly for ulcerative colitis (UC), still requires thorough clinical investigation. Notwithstanding differences in methodologies, current studies demonstrate its potential for inducing remission in UC patients. Therefore, standardized and robust randomized clinical trials (RCTs) are needed to further support its efficacy for managing UC. The aim of the second Rome Consensus Conference was to address gaps and uncertainties identified in previous research regarding FMT and to offer a robust framework for future studies applied to the treatment of UC.

**Methods:**

Global experts in the field of clinical IBD, mucosal immunology, and microbiology (*N* = 48) gathered to address the need for standardized clinical trials in FMT investigation. The group focused on key issues, such as stool donation, donor selection, characterization of fecal biomass, potential administration routes, as well as the process of induction, maintenance, and endpoint readouts.

**Results and Conclusions:**

The consensus achieved during this conference established standardization of methods and protocols to enhance the current quality of research, with the aim of eventual implementation of FMT in managing UC and the ultimate goal of improving patient outcomes.

**Key Messages**

- **What is already known?**

In previous randomized controlled trials, fecal microbiota transplantation (FMT) emerged as a possible and promising therapeutic option for mild to moderate ulcerative colitis (UC).

- **What is new here?**

The results of this consensus conference provide guidance for the design of future clinical trials implementing FMT for managing UC.

- **How can this study help patient care?**

Defining robust and consistent evidence-based information on the efficacy, safety, and mechanism(s) of action in regard to FMT will promote its implementation as a potential therapeutic option in routine clinical practice for managing patients living with UC.

## Introduction

Following its approval for the treatment of *Clostridioides difficile* infection, fecal microbiota transplantation (FMT) has emerged as a potential therapeutic intervention for ulcerative colitis (UC), a condition characterized by chronic relapsing inflammation of the gastrointestinal tract with complex etiopathogenesis.^1^ Despite the potential benefits of FMT, its application for the management of UC remains a frontier topic, requiring rigorous clinical investigation to elucidate its efficacy, safety, and optimal protocols.^2^ Notably, FMT has shown promising results in UC, uncertain outcomes in post-colectomy ileoanal pouchitis, and rather limited efficacy in Crohn’s disease (CD). Different methodologies with regard to mode and frequency of administration, assessment of primary outcome, definitions of outcomes, and choice of comparator were applied in these studies. Moreover, a clear assessment of the benefit/risk and benefit/cost ratios for patients and stakeholders compared to other available therapies is lacking. Therefore, to minimize variations in results and facilitate cross-study comparisons, there is a need for standardized and methodologically robust randomized clinical trials (RCTs) to better understand the role of FMT in inflammatory bowel disease (IBD) treatment, especially in the setting of UC.^1^

**Figure 1.**
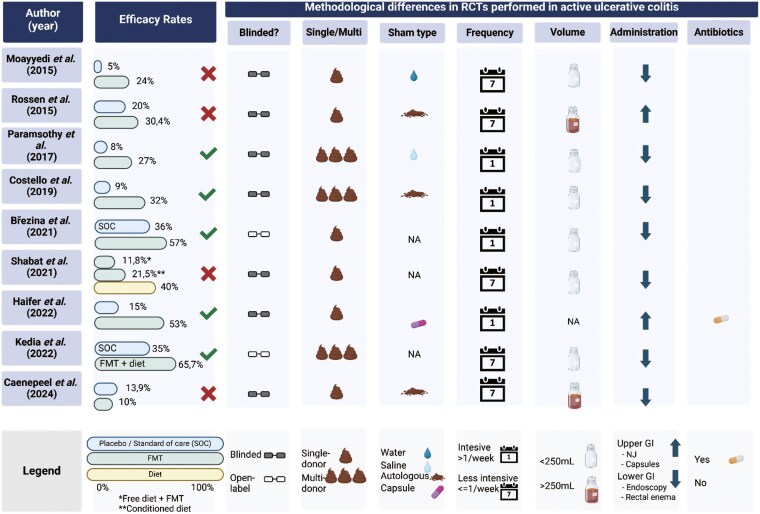
Summary of RCTs conducted, evaluating FMT in active UC. The figure summarizes the efficacy rates and methodologies of randomized controlled trials in terms of clinical remission outcomes. Most studies utilized single donors, except for Paramsothy et al., Costello et al., and Kedia et al., who employed multiple donors. Haifer is the only study that incorporated antibiotic preconditioning. Variations in placebo types, frequency of fecal administration, and fecal volume are also highlighted. Abbreviations: FMT, fecal microbiota transplantation; NJ, nasojejunal tube; RCT, randomized clinical trial; SOC, standard of care; UC, ulcerative colitis.

The second Rome Consensus Conference follows our first Rome Conference, which provided a comprehensive review of the state of the art in FMT for IBD.^1^ This conference brought together leading experts from various specialties from around the globe to agree on key aspects of FMT trial design for UC. The consensus achieved here seeks to address the gaps and ambiguities identified in previous research (Figure 1), offering a robust framework for future studies. Several pivotal issues were discussed, including stool donation, donors’ section, fecal biomass characterization, and administration route. Insights on advisable trial design were also discussed.

By standardizing methodologies and defining reproducible protocols, we expect to facilitate high-quality research that can definitively establish the positioning of FMT in the management of UC. This guidance is therefore intended to drive future research, ultimately improving patient outcomes.

## Methods

To develop a comprehensive guidance for future FMT trials in UC, we assembled a panel of 48 international experts with established expertise in this field, ensuring a multidisciplinary composition. The panel of experts includes gastroenterologists, endoscopists, microbiologists, trialists, pharmacologists, biotechnologists, methodologists, and bio-informaticians. Many participants had previously contributed to the first Rome Consensus Conference. Additional participants expressed their interest and willingness to contribute to this second consensus conference following the publication of the first consensus. Given the collaborative and inclusive nature of this initiative, no exclusion filters were applied. Instead, participation was encouraged among those who demonstrated expertise to advance the field, ensuring a comprehensive and multidisciplinary representation.

An initial set of questions and key discussion points was developed by a core group of authors (F.S. and L.L.). These key issues were largely derived from the previously conducted international Rome consensus conference on gut microbiota and fecal FMT in IBD.^1^ In July 2023, the panel of experts met in Rome to: (1) Review the available data on FMT in IBD patients, focusing on existing quality of evidence and gaps in methodology; (2) discuss, formulate, and agree on strategies for future FMT trials in IBD culminating in a preliminary vote on a set of 33 questions. Following the initial discussions, a structured consensus-building process was implemented. This process included partially rephrasing the preliminary questions to finalize the set of questions and 3 rounds of online voting according to Delphi methodology. Each statement was rigorously evaluated, and those receiving at least 80% agreement from the experts are included in the final guidance document unless stated otherwise.

Consensus was achieved if at least 80% of respondents expressed agreement regarding each statement. Statements that did not pass this threshold were revised and rated again in further rounds of voting until consensus was reached. Panel experts gathered in Rome on July 8, 2023 for refinement and final approval of the overall statements. An overview of all evaluated statements, highlighting those who achieved consensus in every round of voting is included in the Appendix. After achieving consensus, a first draft of the paper was written, incorporating statements and recommendations that achieved an agreement. This draft was then shared with all the authors for thorough review and revision. Each author provided detailed feedback and corrections, ensuring that the final document accurately represented the collective expertise and consensus of the group.

## Results

### Statements

#### General points

- *Despite available information derived from previously performed RCTs, a multicenter placebo-controlled FMT trial for the treatment of UC is still needed. [Agreement: 89.7%]*

The current body of literature on FMT in UC is mainly comprised of monocentric RCTs that focus on the induction of remission in patients with active UC.^3^ While these studies have provided valuable insights, significant knowledge gaps remain. There is a need for further clarification on patient profiling, optimal donor characteristics, and methods of FMT administration. Additionally, the lack of multicenter trials limits the general applicability of findings and the ability to standardize treatment protocols across different settings. Conducting a well-designed multicenter placebo-controlled FMT trial would address these issues, offering more robust and generalizable data that can guide clinical practice and future research.

- *The rationale for performing a multicenter trial is to define new clinical guidelines for FMT in IBD. [Agreement: 82.8%]*
- *The rationale for performing a multicenter trial is to delve deeper into understanding pathogenic mechanisms underlying IBD. [Agreement: 86.2%]*
- *The rationale for performing a multicenter trial is to identify appropriate IBD patient candidates for FMT. [Agreement: 93.1%]*
- *The rationale for performing a multicenter trial is to more precisely determine the magnitude of impact of utilizing FMT in UC. [Agreement: 89.7%]*

Conducting multicenter trials for FMT in UC is crucial for several reasons. Firstly, multicenter trials can establish or strengthen clinical guidelines by encompassing diverse patient populations and treatment settings, thereby increasing the reliability and the translational relevance of the results into clinical practice. Secondly, these trials can provide valuable insights into the pathogenetic mechanisms of IBD, unveil new therapeutic targets, and improve our understanding of disease pathogenesis. Thirdly, multicenter trials are essential to identify the most suitable candidates for FMT and will facilitate treatment toward personalized medicine approaches, according to patient-specific factors. Lastly, these trials can accurately determine the magnitude of therapeutic impact and safety of FMT, the preference of patients, and the overall cost/benefit effects. Table 1 summarizes the ongoing trials using FMT in patients with UC.

**Table 1.** Active trials of FMT in UC.

Study	Location
FMT in Patients With Recurrent CDI and Ulcerative Colitis: Single Infusion vs Sequential Approach	Rome, IT
Transfer of Feces in Ulcerative Colitis 2 (TURN2)	Amsterdam, NL
Study to Evaluate the Fecal Microbiota Transplantation (FMT) in the Treatment of Ulcerative Colitis,	Houston, USA
Superdonor FMT in Patients With Ulcerative Colitis	Rome, IT
Fecal Microbiota Transplantation in Pediatric Ulcerative Colitis (UC)	Wuhan, CH
LFMT vs Placebo in New Biologic Start for Ulcerative Colitis	Edmonton, CA
Oral Fecal Microbiota Transplantation in Pediatric Ulcerative Colitis	Paris, FR
Screening Donors, Fecal Microbiota Transplant Program in Ulcerative Colitis	Hamilton, CA
Safety and Efficacy of Capsule FMT in Treatment-naïve Patients With Newly Diagnosed Chronic Inflammatory Diseases	Odense, DK
Fecal Microbiota Transplantation and Newly Diagnosed Ulcerative Colitis (UC)	Turku, FI
A Multicenter Clinical Trial: Efficacy, Safety of Fecal Microbiota Transplantation for Inflammatory Bowel Disease	Xiamen, CH
Evaluation of the Safety and Efficacy of Lyophilized Fecal Microbiota Transplantation Administered Orally for Prevention of Relapse or Intestinal Inflammation in Adults With Ulcerative Colitis	Houston, USA
Combination Therapy With Fecal Microbiota Transplantation and Vedolizumab for Induction of Ulcerative Colitis	Hamilton, CA
Safety and Efficacy of Fecal Microbiota Transplantation	Hong Kong, HK
Transfer of Frozen Encapsulated Multidonor Stool Filtrate for Active Ulcerative Colitis	Germany
Fecal Microbiota Transplantation for Ulcerative Colitis	Guangzhou, CH
Impact of Fecal Microbiota Transplantation in Ulcerative Colitis	Paris, FR
Fecal Microbiome Transplant	Philadelphia, USA
Standardized Fecal Microbiota Transplantation for Ulcerative Colitis	Nanjing, CH

In the field of *Clostridioides difficile* infection, multicenter studies have consistently yielded positive results. These studies have facilitated the broader dissemination of FMT to specific patient populations, including pediatric patients with particular comorbidities.^4,5^ Inspired by these successes, applying multicenter trial methodologies to FMT in UC could similarly drive significant advancements.

#### Donors

- *Donors should receive complete anamnestic investigation, accurate clinical examination, blood tests, and stool testing, as required for standard FMT donor biobanking. [Agreement: 100%]*

The protocol for screening of FMT donors has been previously described in published guidelines.^6,7^ In brief, preventing the potential spread of infectious illnesses is the fundamental priority behind donor screening for FMT. In several RCTs, including those with UC patients,^8–13^ blood and stool assessments required for FMT screening in cases of *C. difficile* infections have been shown to be safe. A preliminary evaluation should be performed, excluding donors with infectious, gastrointestinal, metabolic, and neurological diseases; furthermore, medications affecting the composition of the gut microbiome, fore example, use of probiotics, should be monitored. To this end, blood and stool tests must be performed and an accurate medical history must be collected at screening; donors should also be reevaluated regularly, depending on the duration of the donation period (eg, weekly), and pre-donation health record should be filled out on the day of donation.^1,7^ Additionally, 3 weeks post-donation, a health record should be collected in order to exclude the occurrence of harmful events.^14^ Such screening methods have provided safe approaches, yet can only be combined with frozen or oral capsule FMT use.^1^ More recent trends also suggest collecting questionnaires regarding dietary habits, physical activity, and specifically addressing risk factors for colonization with multidrug-resistant organisms.^14^

- *A unique donor can donate to different patients. [Agreement: 89.7%]*

Allowing a unique donor to donate to multiple patients can significantly streamline the process of FMT and enhance its feasibility and cost-effectiveness. This approach leverages rigorous screening and preparation of a single high-quality donor sample, ensuring consistency in the microbiota being transplanted. This approach also reduces the logistical burden of identifying and screening new donors for each patient recipient. Importantly, it has generally been accepted to perform single-donor FMTs, whereas multi or pooled FMTs are considered less favorable in order to maintain ecological communities and allow for the identification of specific strains associated with success or failure. However, it should be noted that the effectiveness of single-donor FMTs has only been clearly shown in one double-blind RCT.^9^ Nevertheless, the expert group agrees that single-donor strategies should not be abandoned, as this approach was perceived to be essential for identifying donor characteristics linked to eubiosis restoration and clinical remission.

- *Feces from multiple donors are not recommended for a multicenter trial, based on current legislation. [Agreement: 100%; Statement was formulated and approved during the final common discussion]*

Mixing different donors negatively impacts the quality of the FMT process. First, mixing 2 or more balanced microbiota does not guarantee the generation of a new, balanced ecosystem. Additionally, this practice increases the risk of communicable diseases and complicates the tracing of infection sources in case of adverse events. Lastly, it is challenging to identify factors contributing to better or worse FMT engraftment.^6^

- *For any enrolled patient, an adequate amount of feces for FMT should be available for the entire duration of the protocol. [Agreement: 89.7%]*

Ensuring an adequate supply of fecal material from the same donor for the entire duration of an FMT protocol is crucial for maintaining consistency and efficacy in treatment. This approach minimizes the variability in microbiome composition that can occur with different donor batches and ensures that patients receive a uniform therapeutic intervention. It also helps in avoiding treatment interruptions, which could compromise study outcomes and overall patient health. Proper planning and storage of fecal material, including cryopreservation, are essential to meet this requirement and ensure the success of FMT trials.

#### Stool donation and FMT preparation

- *Stool donation should not be executed on the same day of the FMT, and donor stool should be procured from an FMT biobank. [Agreement: 84%]*

To ensure safety and standardization, donors should be thoroughly screened, according to established guidelines.^1,7^ Consequently, stool donations should not be immediately processed after collection, allowing sufficient time for comprehensive analyses and screening of donor samples. Additionally, selecting donors from an internal stool bank within an FMT center, or well-structured external (eg, national) biobanks where strict criteria for quality control and standards are met, ensures standardized processing.^6^

- *Frozen feces should be used for FMT trials. [Agreement: 91.7%]*

The organizational and qualitative requirements of stool banks have been thoroughly addressed in previous consensus conferences.^6,15^ Utilizing a frozen feces-based FMT approach is essential for standardizing the process and ensuring prompt availability of stool samples, eliminating delays related to new screenings. Collected fecal suspensions can be stored up to 2 years before being discarded.^6^

#### Fecal biomass characterization

- *Donor feces for FMT should be assessed for microbiome composition prior to being used for trial. [Agreement: 84%]*

Together with pathogen screening, assessing the microbiome composition of donor feces is crucial for research-focused FMT studies. In a double-arm clinical trial design (unsupervised FMT vs placebo), this detailed characterization may not be strictly necessary, as the primary objective is to compare clinical outcomes without extensive microbiota profiling. However, for a more research-oriented approach with a 3-arm design (supervised FMT vs unsupervised FMT vs placebo), prior assessment of fecal microbiota composition is essential. Supervised FMT includes the full characterization of fecal biomass, including alpha- and beta-diversity and potentially, the relative abundance of specific strains associated with proven health benefits (eg, *Faecalibacterium prausnitzii*^16^). Additionally, a high abundance of strains associated with FMT failure (eg, *Proteobacteria*, *Fusobacteria*, and *Lactobacilli*^17^) could be discarded. In addition to microbiome profiling, future studies are encouraged to explore the metabolite, for example, bile acids, virome, and mycobiome composition as potential modulators of FMT efficacy.^18,19^ Nevertheless, their role in shaping gut homeostasis and influencing host responses warrants further investigation. This approach would enable a deeper understanding of the correlation between specific microbial communities and clinical outcomes, thereby elucidating the possible mechanisms behind FMT efficacy.

#### Administration route

- *The most suitable route of administration of donor feces in an induction trial is via colonoscopy and/or enemas. [Agreement: 76.0%]*

In *C. difficile* infections, the administration of FMT via the lower gastrointestinal (GI) tract was shown to be more efficacious.^20^ Although a meta-analysis on FMT in IBD has shown no significant difference in efficacy for administration via upper or lower GI tracts, with respective odds ratios (ORs) (95% CI) of 3.11 (0.87-11.12) vs 4.90 (2.14-11.21), initial administration of donor feces via colonoscopy is considered the most suitable route for FMT in induction trials. This method provides a direct delivery to the colon, where it can have the greatest immediate and highest impact. Furthermore, this route for the induction phase was favored,^9^ with a positive impact on clinical and endoscopic outcomes (OR = 4.90, *P* = .002).^3^ Conversely, weekly administration of 50 mL by enema for a 6-week period was the protocol followed for the first RCT in UC.^21^ While endoscopy can be useful to assess endoscopic disease activity at baseline, repeated endoscopic procedures in a short period of time may generate ethical issues and should be carefully considered in the trial design process. In accordance with previous clinical guidance for FMT, recipients should receive conventional bowel lavage with polyethylene glycol before FMT administration via colonoscopy.^7^

Although this statement did not reach 80% approval by the experts (76%), the authors have decided to include it in the results in order to ensure a thorough discussion and provide complete guidance on FMT administration strategies in different phases of treatment. From a clinical practice perspective, the choice should consider disease location, the need for endoscopic assessment, and cost-effectiveness. From a trial design perspective, resolving this issue will only require head-to-head comparisons of different routes to evaluate efficacy, safety, and patient preferences.

- *The most suitable route of administration of donor feces for FMT in a maintenance trial is via capsules or via enema. [Agreement: 94%]*

Currently, RCTs evaluating FMT for long-term maintenance of remission are lacking. The limited studies that have been performed used single dose or 8-weekly administrations via colonoscopy.^22,23^

Although FMT administration via colonoscopy for the maintenance phase is well tolerated and guarantees an adequate delivery of healthy microbiota, for specific maintenance trials, administration of FMT via oral capsules containing lyophilized feces or rectal enema is considered the most suitable approach by the expert panel. These methods are less invasive and more convenient compared to colonoscopy, and more accepted by patients for long-term use. Furthermore, both enemas and capsules facilitate the administration of a sham placebo (discussed later). In the case of a large international trial, the expert panel agrees that, due to feasibility reasons, safety regulations in different countries, and the need to evaluate the viability of lyophilized feces, rectal enemas are preferred over oral capsules.^15^

- *Sham placebos (prepared with colorants and/or masked according to current legislation) should be administered as a control. [Agreement: 96.4%]*

A sham placebo, designed to mimic the appearance and consistency of donor feces, helps maintain the integrity of the study by ensuring a blinded approach and reducing the risk of placebo effects. In the past, water,^8^ saline^10^ solutions, and autologous solutions have been utilized. Specifically, remission of disease has also been observed following the administration of autologous feces,^11–13^ which could confound results and undermine the validity of the trial. In fact, this could be the possible underlying reason that 2 out of 3 trials^12,13^ applying autologous FMT might have failed—both used an anaerobic workflow for the autologous FMT preparation, whereas the third^11^ did not. Thus, these specific methods might also play a role, and should be standardized, in future trials. Therefore, this expert panel agrees that a colored and odored saline sham control might be preferable.

- *FMT by enemas for the maintenance scheme should be administered, preferably every 4 weeks, although other schemes could be acceptable, as long as uniformity and homogeneity in the trial design are provided. [Agreement: 92.6%]*

In a randomized pilot trial, Sood et al. showed that maintenance FMTs by colonoscopy every 8 weeks in patients in remission for a total duration of 48 weeks^22^ could help to sustain clinical, endoscopic, and histological remission for UC patients. Unfortunately, very recent evidence shows that a single FMT dose applied by colonoscopy did not impact the maintenance of remission.^23^ Therefore, the expert group proposes a treatment regimen with FMT maintenance using rectal enemas or oral capsules every 4 weeks, thus monthly.

- *The end of the maintenance phase can be set at week 24 or week 52, as long as consistency and homogeneity in the trial design are ensured. [Agreement: 92.6%]*

In previous studies evaluating long-term maintenance therapy (>12 weeks), the endpoint was set at either 48 or 52 weeks.^13^ Taking this into account, the expert panel proposes to set the endpoint at least at 24 weeks; however, either 52 weeks or 1 year is preferred, as recommended by the regulatory organizations.

#### Patient characteristics

- *Adult patients (age 18-80) should be considered eligible to participate in the FMT trial if suffering from mild to moderate UC (including proctitis), and under stable therapy. [Agreement: 80%]*

Consistent with previous studies, the expert panel agrees that adult patients with mild to moderate UC (modified Mayo Score 4-6) should be allowed to receive FMT trial therapy. Additionally, recent work by Caenepeel et al. ^13^ highlighted the importance of patient selection in FMT trials in agreement with previous findings^24^ and therefore, further patient selection is required based on disease severity. Additionally, patients displaying proctitis were often excluded from previous FMT trials; however, the expert panels agree that by applying a pragmatic approach, these patients should be included in future trials. Recently, FMT protocols in pediatric populations are being performed and published,^25^ wherein adolescent or pediatric patients under the age of 18 may be included, provided that all legal and regulatory requirements are fully met.

- *The standard of care should always be followed (ie, mesalamine administration). FMT can be provided as an add-on therapy against placebo. [Agreement: 96.6%]*

Mild to moderate UC patients should always be able to receive the standard of care; therefore, FMT trial patients should be randomized over the treatment arms by balancing for the treatment they are currently receiving. Subsequently, the additional effect of FMT on standard of care treatments could be evaluated. Importantly, concomitant antimicrobial or probiotic treatment should not be allowed for the duration of the trial.

- *Eligible patients for FMT trials may include biologic- (or small molecule-) naïve individuals. [Agreement: 85.2%]*

Patient selection has been shown to be important, as in the RESTORE-UC trial no patients on biological treatment reached the primary endpoint. Therefore, FMT should preferably be performed in biologic- or small molecule-naïve patients, as this subpopulation may comprise patients with less severe disease and therefore allow higher efficacy rates for FMT treatment.

- *Patients with a history of prior biologic (or small molecule) exposure may be considered eligible for FMT trials, provided that the trial design ensures uniformity, consistency, and homogeneity. This includes factors, such as the number of prior exposures/failures and the specific biologic drugs previously used. [Agreement: 96.3%]*

Previous biologic or small molecule exposure/failure should not lead to exclusion based on the aforementioned status. However, when including these patients, it is of utmost importance to make sure that their treatment is stable and their disease activity is mild to moderate. Moreover, enrolling patients with previous failure(s) to no more than one class of biologic agents would be preferable. Prior exposure should be recorded at baseline and included as a stratification variable in the randomization process.

- *Next to pathogen screening, patients with UC should undergo assessment of microbiome composition prior to inclusion in the trial. [Agreement: 88%]*

Although not present in all patients, dysbiosis has been associated with UC.^26,27^ Aiming to restore this dysbiosis by FMT therapy, evaluating microbiome composition at baseline is extremely important as low efficacy rates have been observed upon inclusion of a limited number of dysbiotic patients.^13^

#### Efficacy evaluation

- *The primary outcome should be clinical remission. Clinical response, endoscopic response, and endoscopic remission should be secondary outcomes. Donor microbiota engraftment should be a secondary outcome. [Agreement: 88%]*

Although slightly different definitions were used,^3^ previous trials mainly evaluated clinical remission at the primary endpoint.^8–13,24^ Moreover, changes in Mayo Score can be monitored and considered as a secondary endpoint. Additionally, the transfer of donor microbiota was considered a primary endpoint^28^; however, the expert panel agrees that in terms of FMT efficacy in active UC patients, the primary endpoint should be (steroid-free) clinical remission. Subsequently, clinical response, endoscopic remission, and response should be considered as secondary endpoints. Nevertheless, microbiota transfer from donor to patient should also be considered as a secondary endpoint. Furthermore, it is important to keep track of mucosal healing, based on both endoscopic and histologic remission, as defined by the Food and Drug Administration (FDA) and European Medicines Agency (EMA).^29^

- *Patients with UC should be assessed using modified Mayo Scores and modified endoscopy Mayo Scores. [Agreement: 92%]*

In order to provide a standardized method to report patient data, the expert group agrees that modified Mayo Scores and modified endoscopy Mayo Scores should be used. In comparison to traditional Mayo Scores, modified Mayo Scores reduce subjectivity and lead to more reproducible results. Furthermore, it has shown a similar, if not better, correlation with patient outcomes.^30^

- *The primary endpoint of the induction phase can be set at week 8 or week 12, as long as consistency and homogeneity in the trial design are ensured. [Agreement: 100%]*

The primary endpoint of the induction phase can be set at either week 8 or 12, as observed in previous FMT trials for UC.^9–13,31^ During this phase, administration of FMT through colonoscopy and enemas was proposed previously (general points) as a feasible approach. A recent meta-analysis^3^ indicated that there were no differences in efficacy for an intensive (>1 FMT/week) vs less intensive (< or =1 FMT/week) treatment regimen. Establishing 8-12 weeks as a time-point may also allow future data analyses for indirect comparisons with other treatments (ie, network meta-analyses). Accordingly, longer induction phases (>16 weeks), including a placebo arm, are generally not accepted by patients.

- *Open-label FMT could be offered as a therapeutic option after failure. [Agreement: 79.3%]*

In case the patient does not respond at the end of induction, open-label FMT should be proposed upon which the patient would be free to decide whether or not to accept the open-label donor FMT. Moreover, it might be considered to allow non-responders, who received FMT from a healthy donor, to receive a second donor FMT, upon the agreement of the patient.

- *Open-label FMT could be offered as a therapeutic option for responders at the end of the trial. [Agreement: 89.7%]*

In terms of maintenance therapy, and upon completion of the trial, patients could be allowed to continue FMT treatment to maintain remission.

#### Basic and translational considerations

- *Assessment of microbiota modulation through the trial is mandatory. [Agreement: 91.7%]*

The assessment of microbiota modulation within clinical trials is imperative. Understanding modification of the gut microbiota composition, and functionality post-FMT, is crucial for elucidating potential mechanisms underlying treatment efficacy. The 2019 RCT by Paramsothy et al. has shown enrichment of *Eubacterium hallii* and *Roseburia inulinivorans*, as well as increased levels of short-chain fatty acids and secondary bile acids in patients with UC achieving remission after FMT.^32^ Similarly, in a recent open-label protocol performing 3 serial FMTs within 6 weeks, *Parabacteroides, Bacteroides, Faecalibacterium*, and *Akkermansia* were higher in responders at all timepoints.^33^ Future studies should also expand microbiome analyses to include metabolite (eg, bile acids), virome, and mycobiome composition.^34^

- *Translational studies associated with the clinical trial would be preferable. [Agreement: 91.7%]*

Integrating translational studies alongside clinical trials is not just preferable, but essential—although not mandatory to perform prior to the clinical trial. For example, studies using murine or in vitro models of IBD can provide valuable insights into the immunological and microbiological changes induced by FMT. These models allow researchers to investigate the effects of FMT on the gut microbiota, the immune system, and the intestinal epithelium in a controlled environment. Such studies can help to identify key players in the FMT response, including specific bacterial species, metabolites, or immune cells, and inform the design of clinical trials. In addition, translational studies can also involve the analysis of human samples from clinical trials, such as blood, stool, or tissue biopsies. These studies can help to identify biomarkers that predict response to FMT, monitor changes in the gut microbiota and immune system during treatment, and provide insights into the mechanisms of FMT efficacy.

#### Trial design

- *A dual-arm trial design, comparing unsupervised FMT against a sham FMT, represents a feasible and appropriate study design to test the efficacy and safety of FMT. A 3-arm trial design, including supervised FMT, could provide valuable insights but may be considered for subsequent, more comprehensive studies contingent upon the success and findings of this trial. [Agreement: 91.3%]*

Most trials in IBD have exploited a dual-arm trial design^9–13,21,35^ and therefore, are also considered feasible in terms of statistical sensitivity and required number of patients. However, adding a supervised FMT arm in which rigorous donor selection is performed at the microbiota level could provide valuable insights in terms of donor selection and identification of specific microbial communities responsible for FMT efficacy. Unfortunately, in the RESTORE-UC trial, this approach of donor selection was applied in a 2-arm setting, and it was unable to increase efficacy rates. Yet, the negative outcome reported in RESTORE-UC was considered by the authors to be related to patient, rather than donor, characteristics. Additionally, the same study showed that a lower bacterial load at baseline leads to higher remission rates,^13^ indicating that an antibiotic pretreatment might be considered in order to increase engraftment as also observed in a pilot study by Ishikawa et al. ^36^ and in the LOTUS-trial.^9^ Yet, antibiotics followed by placebo might lead to further disruption of the microbiota composition, as an incomplete composition recovery was previously observed.^9^ Therefore, implementing an antibiotic pretreatment should be carefully considered. Other methods for a supervised approach could comprise preclinical evaluation of donor material by in vitro and in vivo methods. Subsequently, upon completion of the trial, these methods can be evaluated in terms of success prediction. When not opting for a supervised arm, a post hoc analysis might provide a compromise—although a large sample size would be required.

Finally, the panel discussed the incorporation of artificial intelligence (AI) tools into future FMT trials. Specifically, the use of federated learning was proposed to facilitate multicenter data analysis while preserving data privacy and security. Such AI-based approaches can improve donor selection processes, optimize microbiota profiling, and enhance the statistical modeling of trial designs by leveraging large datasets without compromising confidentiality.^37,38^ Additionally, AI-driven microbiota analysis may enable predictive modeling to identify biomarkers of treatment response, streamline donor matching, and finally improve therapeutic outcomes.

## Conclusions and Future Perspectives

**Figure 2.**
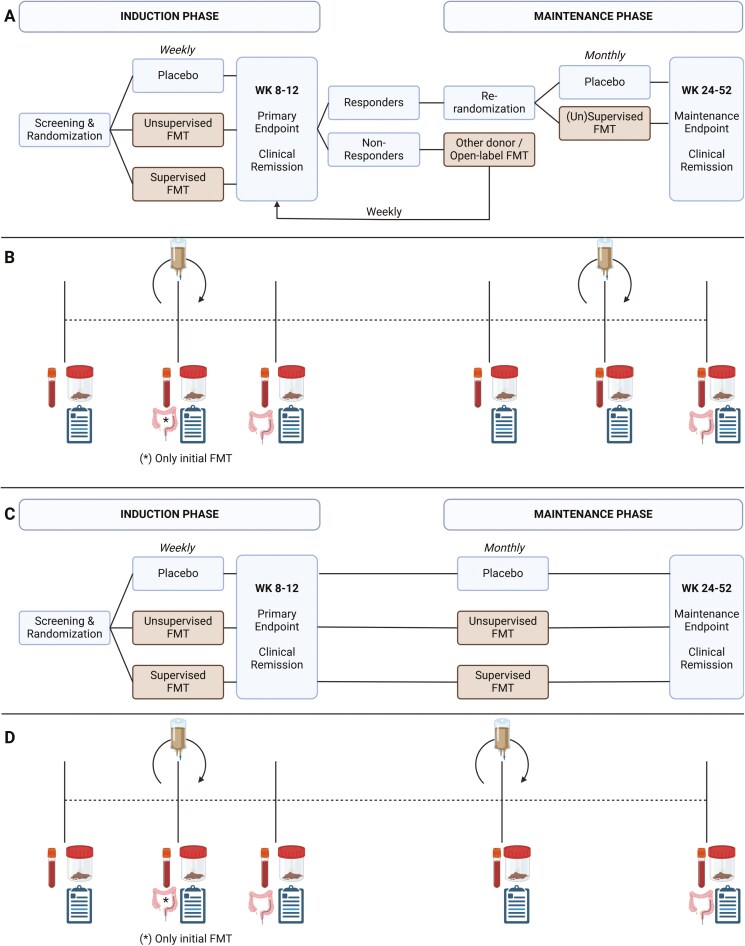
Trial design and proposed sample collection for FMT clinical trial for the treatment of UC. Strategy for (A, B) re-randomization and (C, D) treat-through approach, wherein, respectively, patients could be re-randomized after the induction phase to placebo or (un)supervised FMT or maintained on sham/FMT derived from the same donor. At the different study visits, different samples should be collected including biopsies (baseline and primary endpoint), blood, and fecal samples. Additionally, also dietary and physical activity questionnaires should be collected. Abbreviations: FMT, fecal microbiota transplantation; UC, ulcerative colitis; Wk, week.

Several RCTs have evaluated the efficacy of FMT to induce remission in active UC patients, but conflicting results have been obtained and due to different methods used, results remain challenging to compare. Standardization of methods is required to bring FMT a step closer to a potential future therapeutic option to treat mild to moderate UC patients. The expert panel agreed on several procedures that should be implemented in future trials. Accordingly, the experts propose potential study designs (Figure 2), wherein the induction phase (lasting 8 to12 weeks) should consist of weekly FMTs initially administered by colonoscopy and continued by rectal enemas. The primary endpoint should be one of clinical remission, and non-responders should be offered either open-label donor FMT, changing FMT donor, or provided the option to end the trial. Regarding the maintenance phase, several designs could be considered, including a treat-through design or re-randomization of responder design (eg, same/other donor or placebo) to evaluate the effect of maintenance FMT. Yet, it would be important to exclude bias introduced by cross-over when deciding to change donors. For practical matters, it is therefore crucial to obtain enough single donor material prior to individual patient inclusion. Sham placebo should be administered. Aside from the clinical trial design, laboratory standardization is also required. For this, a standardized protocol detailing how feces are collected and processed (aerobic vs, anaerobic workflow), what dose and volume of FMT are administered (cell count based vs weight based), what defrosting procedure and viability assessments prior to administration are, and what quantitative microbiota profiling will be performed before, during, and after the trial.

Future FMT trials should also rigorously characterize donor microbiota (microbial diversity and taxonomy in particular) and investigate the effects on IBD recipients before and after FMT. For sure, such an investigational design is demanding in terms of costs, resources, and facilities. However, this approach would guarantee countless benefits. In fact, the collected data could be leveraged to maximize FMT efficacy and define the ultimate positioning of FMT in clinical practice. Furthermore, elucidating its mechanisms of action could unveil new possibilities, guiding the pharmaceutical industry in developing microbiome-based consortia. Additionally, evaluating the convenience of FMT compared to standard therapies is mandatory and requires assessing its efficacy, safety, patient benefits, and cost-effectiveness. This specifically involves comparing clinical outcomes, adverse effects, and patient-reported quality of life with those of standard treatments. Additionally, a thorough cost analysis should consider both direct and indirect costs, including work absence and long-term financial impact. In terms of clinical practice costs, it is noteworthy that the implementation of FMT as an add-on therapy could reduce expenses associated with expensive second-level therapies (biologic drugs, small molecules) for patients not fully responding to mesalamine. Finally, ethical implications must be considered. Ethical committee approval should be obtained by all the centers involved in this trial. Patients should receive thorough and ongoing informed consent, ensuring awareness of the possible placebo administration. Despite the potential benefits of FMT, the complexity of trials required to capture these elements poses a significant challenge in determining its overall convenience for patients.

Further enhancements to FMT efficacy should be explored through supportive measures, including donor and recipient diets, bowel preparation, antibiotic pretreatment, and the use of probiotics, prebiotics, synbiotics, and postbiotics. Combination therapy with existing IBD treatments should also be examined. Moreover, the identification of specific microbiota strains that predict FMT success or failure could lead to the development of well-defined single- or multi-strain probiotics,^39^ or targeted treatment strategies such as bacteriophages.^40^ Finally, the implementation of AI-based technologies will improve the quality and reproducibility of data.
